# Refining orthoplastic reconstruction timing and flap selection in Gustilo–Anderson type IIIB/C open tibial fractures

**DOI:** 10.1097/JS9.0000000000003359

**Published:** 2025-09-08

**Authors:** Rui Liu, Yongbing Xiao

**Affiliations:** Department of Orthopaedics, Xiangya Hospital, Central South University, Changsha, China

*Dear Editor*,

We read with great interest the article by Wang *et al*^[1]^, which presents a large-scale, well-executed study on the orthoplastic management of Gustilo–Anderson type IIIB/C open tibial fractures. The prospective design and substantial sample size are particularly commendable, and we would like to offer several comments based on our clinical experience. This correspondence is compliant with the TITAN Guidelines 2025-governing declaration and use of AI^[2]^. We acknowledge that AI-assisted tools (ChatGPT 4o, OpenAI) were used to improve the clarity and grammar of the written language in this letter. No AI tools were used to generate original content, data, or analysis.

We noted that in both cohorts, reconstruction was performed in a staged manner, with soft-tissue coverage delayed to a second operation. Drawing on our clinical experience, we previously conducted a comparative study of emergency (single-stage) versus delayed reconstruction in 394 cases over a 15-year period. Our findings demonstrated significant advantages of early reconstruction, including reduced flap complications and chronic infection, faster bone union and wound healing, shorter hospitalization, and improved functional outcomes^[3]^.

While Wang *et al*’s orthoplastic strategy yielded lower infection rates and better function compared to orthopedic-alone treatment, we believe the study did not fully explore the value of true early, single-stage reconstruction, especially in clean wounds. In our practice, timely coverage using chimeric flaps in the emergency setting allows simultaneous reconstruction of both soft-tissue and bone defects, often avoiding the need for prolonged staged procedures. With modern microsurgical techniques, such one-stage reconstructions are increasingly achievable – even in Gustilo type IIIC injuries – with encouraging outcomes^[4]^.

We also wish to comment on flap selection in soft-tissue reconstruction. The exclusive use of free anterolateral thigh (ALT) flaps may not be optimal for all cases. In smaller or moderate-sized defects, free flap harvesting can be technically challenging due to variability in perforator anatomy, and accurate dissection of small skin paddles is often difficult. In such cases, pedicled flaps are commonly used for soft-tissue coverage of the lower leg. These flaps avoid sacrificing major recipient vessels, as is necessary with free ALT flaps, and offer shorter operative time without the risks associated with microvascular anastomosis^[5]^. Free flaps are more appropriate for moderate-to-large composite defects, and in selected complex cases, combined flaps from multiple donor sites may be warranted^[6]^.

Finally, we acknowledge that the technical proficiency demonstrated by Wang *et al* reflects a strong foundation of orthoplastic training in China. As Dr. Jinbo Tang noted, early and high-volume exposure to microsurgical procedures – particularly in trauma centers – enables Chinese surgeons to develop considerable operative fluency early in their careers. We appreciate the valuable contribution of this work and hope our comments offer further perspective on optimizing surgical timing and technique in the management of complex open tibial fractures.

## Data Availability

Data sharing is not applicable to this article.
